# The Potential Genes Mediate the Pathogenicity of Allogeneic CD4^+^T Cell in aGVHD Mouse Model

**DOI:** 10.1155/2021/9958745

**Published:** 2021-05-07

**Authors:** Zhengyu Yu, Chenchen Qin, Min Cao, Xiaoya He, Hanyun Ren, Huihui Liu

**Affiliations:** Department of Hematology, Peking University First Hospital, Beijing, China

## Abstract

Acute graft-versus-host disease (aGVHD) remains a significant and severe complication of allogeneic hematopoietic stem cell transplantation (allo-HSCT). Due to the occurrence of aGVHD, allo-HSCT significantly increases the mortality rate compared with autologous hematopoietic stem cell transplantation (auto-HSCT). In this study, auto-HSCT and allo-HSCT aGVHD mouse models were built to detect the difference in CD4^+^ lymphocyte in different tissues based on ribonucleic acid sequencing (RNA-Seq) analysis. Clustering analysis, functional annotation, and pathway enrichment analysis were performed on differentially expressed genes (DEGs). The protein-protein interaction (PPI) network was used to find hub genes. CD4^+^T cells were activated by MLR and cytokine stimulation. Cells were sorted out by a flow cell sorter. The selected genes were verified by qRT-PCR, histology, and immunofluorescence staining. The GSE126518 GEO dataset was used to verify the hub genes. Enrichment analysis revealed four immune-related pathways that play an important role in aGVHD, including immunoregulatory interactions between a lymphoid and a nonlymphoid cell, chemokine receptors binding chemokines, cytokine and cytokine receptor interaction, and the chemokine signaling pathway. At the same time, with the PPI network, 11 novel hub genes that were most likely to participate in immunoregulation in aGVHD were identified, which were further validated by qRT-PCR and the GSE126518 dataset. Besides, the protein expression level of Cxcl7 was consistent with the sequencing results. In summary, this study revealed that immunoregulation-related DEGs and pathways played a vital role in the onset of aGVHD. These findings may provide some new clues for probing the pathogenesis and treatment of aGVHD.

## 1. Introduction

Allogeneic hematopoietic stem cell transplantation (allo-HSCT) is an effective treatment for various haematological diseases wherein blood cells are unavailable in unmodified autologous transplants. Acute graft-versus-host disease (aGVHD) is one of the significant complications of allo-HSCT. Despite considerable achievements in the treatment of aGVHD, conventional clinical treatments of aGVHD are still targeted at the entire immune system, which not only increases the risk of serious infection but also leaves some patients at risk of developing aGVHD [1]. The occurrence of aGVHD can be divided into three stages according to its sequence. Firstly, preconditioning regimens such as radiotherapy and chemotherapy as well as infection cause epithelial cell damage and an inflammatory environment, while the expression of the MHC antigen in the recipient initiates the recognition of donor T cells. Secondly, the interactions between T cell receptors, antigen major histocompatibility complexes (anti-MHC), costimulatory molecules, and cytokines activate the donor T cells at an early stage. In this stage, T cells in the transplant are activated under the stimulation of an inflammatory environment and an allogeneic antigen, clonally proliferate, and finally differentiate into helper T cells (CD4^+^T cells) or cytotoxic T cells (CD8^+^T cells). Lastly, the activated effector T cells migrate to target tissues and recruit more effector cells, and various cytokines are released abnormally to act on effector cells resulting in damages to the lung, gut, and liver [2].

Alloreactive T cells proliferate and differentiate into subsets of T cells, including Th1, Th2, Th17, and others in peripheral lymphoid tissues, which are considered as the major detrimental factors for the pathogenesis of aGVHD [3]. IFN-*γ*-secreting Th1 cells are considered the key factor in the onset and progression of aGVHD [4]. Among all T cells, donor-derived CD4^+^T cells and secreted cytokines are particularly vital in the pathogenesis and regulation of aGVHD. Studies have shown that the regulation of donor-derived CD4^+^T cells and the production of some critical cytokines (such as IFN-*γ*) could affect the development of aGVHD [5]. CD4^+^T cells have been studied as the potential treatment targets for GVHD in a large number of clinical trials [4, 6]. A clinical study, however, found that peripheral blood stem cell (PBSC) graft containing more mature T cells did not increase the incidence and severity of aGVHD [7]. Some studies found that the infiltration of immunomodulatory Treg cells in the intestine, skin, and stomach was different [8–10]. Meanwhile, we found a phenomenon that the infiltration of activated effector CD4^+^T cells in these tissues was different in the aGVHD mouse model in this study. The exact character of activated effector CD4^+^T cells in the development of aGVHD remains unclear.

As a transcriptome sequencing technique, ribonucleic acid sequencing (RNA-Seq) is used to study alternative splicing and other forms of alternative isoform expression [11]. There are, however, few reports on the mechanism of aGVHD-related target organ damage by RNA-Seq. In this study, using RNA-Seq, we first analyzed the gene expressing profiles of CD4^+^T cells from the spleen, lung, and liver tissues sampled in auto-HSCT and allo-HSCT recipient mice and identified the differentially expressed genes (DEGs) to characterize CD4^+^T cells in aGVHD.

## 2. Materials and Methods

### 2.1. Mice

Female C57BL/6 (H-2b) and male Balb/c (H-2d) mice were purchased from the Animal Experiment Center of Peking University Health Science Center. C57BL/6 mice, 8-10 weeks of age with a body weight of 19–21 g each, were donors of bone marrow hematopoietic stem cells and spleen lymphocytes. Balb/c mice, 10-12 weeks of age with a body weight of 20–23 g each, were recipients. After transplantation, mice were fed with water containing gentamicin for seven days. Mice were bred under specific pathogen-free (SPF) conditions at the Animal Breeding Center of Peking University Health Science Center. All experimental protocols were approved by the Animal Care and Use Committee of Peking University Health Science Center. The study design is illustrated in Figure S1 for the experimental and analysis scheme.

### 2.2. aGVHD Induction in a Murine Model

The method of aGVHD induction using radiation-exposed mice and allogeneic bone marrow transplants was described previously [12–14]. Briefly, bone marrow cells were acquired from C57BL/6 mice. Splenocytes were obtained from C57BL/6 and Balb/c mice by Ficoll gradient centrifugation. Auto-HSCT and allo-HSCT recipient Balb/c mice were irradiated with 7.5 Gy of total body irradiation (TBI). Autogeneic hematopoietic stem cell transplant (auto-HSCT) Balb/c recipient mice were intravenously injected with 1 × 10^7^ bone marrow (BM) cells. Allo-HSCT Balb/c recipient mice were intravenously injected with 1 × 10^7^ BM cells and 1 × 10^7^ splenic mononuclear cells. Balb/c recipient mouse were then evaluated based on a clinical score, including activity, skin integrity, weight loss, posture, and fur texture every other day for one month.

### 2.3. T Lymphocyte Enrichment

CD4^+^T cells were negatively selected from the liver, lung, and spleen of Balb/c recipient mice using a mouse CD4^+^T cell isolation kit (BioLegend, San Diego, CA, USA) according to the manufacturer's instructions. CD4^+^ lymphocytes were prepared at a concentration of 5 × 10^6^/mL. The purity of the enriched populations was >90% as assessed by a flow cytometry.

### 2.4. Antibodies and Flow Cytometry

The following anti-mouse antibodies from BioLegend (Cal: 100421, 103043, and 104411; US) were used in flow cytometry: CD4-PE/Cy7, CD44-BV510, and CD62L-APC. Spleens, lungs, and livers were excised and gently pressed through a cell strainer (70 *μ*m) on the seventh day after transplantation. Infiltrating lymphocytes in the liver and lung were isolated using Percoll (Living, Beijing, China). Single-cell suspensions were blocked by incubating with anti-Fc receptor antibody for 10 minutes on ice. Cells were then labelled with fluorescently conjugated antibodies at 4°C for 30 min, followed by washing with cold PBS twice before performing membrane molecule analysis using a flow cytometer. Cells were sorted out using BD FACSymphony S6, and gating was done using the BD FACSDiva Software (BD Biosciences).

### 2.5. RNA Preparation

Total RNA was extracted from CD4^+^ T cells isolated from the liver, lung, and spleen using the TRIzol Reagent (Thermo Fisher Scientific, Waltham, MA, USA) and the Qubit® RNA Assay Kit in a Qubit® 2.0 Fluorometer (Life Technologies, CA, USA). RNase-free DNase I (Invitrogen, Carlsbad, CA, USA) was used to digest potential genomic deoxyribonucleic acid (DNA). The digested products were then purified using magnetic beads (Axygen, Union City, CA, USA) to eliminate residual DNA and DNase. The concentrate of the extracted RNA was analyzed with a Qubit® RNA Assay Kit in Qubit®2.0 Fluorometer (Life Technologies, CA, USA). RNA purity and integrity were assessed using a NanoPhotometer® spectrophotometer (Implen, CA, USA) and an RNA 6000 Nano Assay Kit of the Agilent 2100 Bioanalyzer system (Agilent Technologies, CA, USA). Total RNA of all samples had a concentration ≥ 200 ng/mL, a mass ≥ 10 mg, and an RNA integrity number (RIN) ≥ 8.0.

### 2.6. Complementary DNA (cDNA) Library Construction

The poly-T oligo-attached magnetic beads were used to purify mRNA from the total RNA. Fragmentations were carried out using divalent cations under elevated temperatures in a First Strand Synthesis Reaction Buffer (5x). The cleaved RNA fragments served as the templates for the synthesis of the first-strand cDNA using a random hexamer primer and an M-MuLV reverse transcriptase (RNaseH-). The second strand cDNA was synthesized using DNA polymerase I and RNase H. The double-stranded cDNA was then degraded to blunt ends using an exonuclease/polymerase. After adding a terminal A at the 3′ ends in DNA fragments, library fragments were purified using the AMPure XP system and cDNA fragments of 250~300 bp in length were selected (Beckman Coulter, Beverly, USA). The sequencing adaptors and the cDNA fragments were mixed and ligated at 37°C for 15 min. A polymerase chain reaction (PCR) was performed with the Phusion High-Fidelity DNA Polymerase, universal PCR primers, and an index (X) primer. The integrity and size of the product were verified on an Agilent 2100 Bioanalyzer (Agilent Technologies, Santa Clara, CA, USA).

### 2.7. RNA-Seq Data Processing and Identification of DEGs

cDNA libraries of CD4^+^ T cells from the auto-HSCT and allo-HSCT models were sequenced at Novogene Bioinformatics Technology Co. (Beijing, China). Paired-end sequencing was performed on an Illumina Hiseq™ 4000 platform (Illumina, San Diego, CA, USA). FastQC software was used to preprocess raw reads to remove reads containing PCR duplicates, adapters, Ploy-N, and low-quality reads. In this step, Q20, Q30, and GC contents of the clean reads were calculated. All the subsequent analyses were based on clean data with high quality.

Gene model annotation files and the reference genome were downloaded from the genome website (http://apr2018.archive.ensembl.org/Mus_musculus/Info/Annotation). Hisat2 v2.0.5 was used to build the index of the reference genome and to match paired-end clean reads with the reference genome. The sequencing process possibly has machine errors, and the inspection of the error rate can reflect the quality of sequencing data. The error rate was obtained through the conversion of the Phred score of the sequencing base through the formula Qphred = −10log10(*e*), where *E* is the base error rate [15].

Fragments per kilobase per million reads (FPKM) was used to calculate the transcript expression levels [16]. DESeq2 R package (1.16.1) was used to identify DEGs in CD4^+^T cells from different organs between auto- and allo-HSCT mice [17].

According to the manufacturer's instructions, the clustering of the index-coded samples was carried out on a cBot Cluster Generation System by TruSeq PE Cluster Kit v3-cBot-HS (Illumina). The aggregations of all groups' DEGs were taken as the DEG sets. In the multigroup experiments, the clustering analysis of the DEG sets was performed to gather genes with similar expression patterns.

### 2.8. GO and Pathway Enrichment Analysis

All DEGs were mapped to terms in the Gene Ontology (GO) databases for functional enrichment analysis. *p*adj < 0.05 was considered significantly enriched. GO terms were classified into three subgroups: biological process (BP), cellular component (CC), and molecular function (MF). Pathways with *p*adj < 0.05 for DEGs were further annotated by the Kyoto Encyclopedia of Genes and Genomes (KEGG) and the Reactome automatic annotation server [18, 19]. Genome enrichment analysis of DEGs in immune-related pathways was performed using Gene Set Enrichment Analysis (GSEA) [20].

### 2.9. PPI Network Construction Analysis

STRING (Search Tool for the Retrieval of Interacting Genes) was applied to predict the protein-protein interaction (PPI) network for DEGs [21]. The Cytoscape software was used to visualize and analyze the PPI network (confidence score ≥ 0.4; maximum number of interactors = 0) [22]. The cytoHubba plugin from the Cytoscape was applied to look for hub genes according to the Maximal Clique Centrality (MCC) [23]. The intuition behind MCC is that essential proteins tend to be clustered in a yeast protein-protein interaction network. The Molecular Complex Detection (MCODE) plugin was applied to make analysis of the gene network functional module. The method is based on vertex weighting based on local neighborhood density and outward traversal of local dense seed protein, and the dense regions are separated according to the given parameters [24].

### 2.10. Validation of Gene Expression by qRT-PCR

Real-time quantitative reverse transcription polymerase chain reaction (qRT-PCR) was performed to validate the expression levels of 11 DEGs related to immunoregulation. The murine gene *GAPDH* served as an endogenous reference. The primers for all genes are presented in Table 1. Total RNA was extracted and used as a template to be reversely transcribed into single-stranded cDNA using a Revert Aid First-Strand cDNA Synthesis Kit (Thermo Fisher, Waltham, MA, USA). The ABI Prism 7500 PCR system was used for real-time monitoring of the SYBR Green dye (Life Technologies, Foster City, CA, USA) integrated into PCR products. Reactions were carried out in the following conditions: initial denaturation at 50°C for 2 min and then 95°C for 10 min, followed by 40 cycles of reaction at 95°C for 15 s and 60°C for 60 s. Each sample had three technical replicates (3 independent wells with the same template) to ensure the precision of the quantification. The 2^−ΔΔCt^ method was used for gene expression.

### 2.11. Cell Stimulation and Expansion

T cells (5 × 10^5^/mL) from the spleens of Balb/c or C57BL/6 mice, separated by the CD4^+^T cells negatively selected through magnetic cell sorting, were stimulated with anti-CD3/CD28 antibodies coated on 96-hole round bottom plates at 500 *μ*L with 2 *μ*g/mL each (PeproTech, USA) for 48 hours.

### 2.12. Mixed Lymphocyte Reaction

Spleens were isolated from the mice, ground into individual cells, and treated with 50 *μ*g/mL mitomycin C (Sigma-Aldrich, USA) for 30 min at 37°C. Washed three times by PBS, responders (0.5 × 10^6^ C57BL/6 CD4^+^T cells) and stimulators (0.2 × 10^6^ Balb/c spleen cells) were cocultured in 200 *μ*L of RPMI 1640 culture medium containing 10% FBS, 100 *μ*g/mL streptomycin, and 100 *μ*g/mL penicillin. Within 72 hours, the cells were collected and tested for T cell activation. Activated C57BL/6 CD4^+^T cells (H2KB^+^CD4^+^CD44^+^) were separated and sorted out by fluorescence-activated cell sorting (FACS).

### 2.13. Histological Research

Mice in each group were sacrificed on the 7th day after transplantation. The liver, lung, and spleen tissues were fixed in 10% buffered formalin and embedded in paraffin blocks for subsequent hematoxylin and eosin staining. Histologic changes were graded and scored by a pathologist blinded to the clinical status of the mice. Four items were assessed as described previously [25]. To determine the infiltration of T cells in the tissues of post-HSCT mice, immunohistochemical analysis was performed on the liver, lung, and spleen tissues of recipient mice at 7 days posttransplantation, stained with the primary antibodies anti-CD4 (1 : 20, Servicebio, Wuhan, China) and anti-Cxcl7 (1 : 20, Abcam, Cambridge, UK) as well as the appropriate horseradish peroxidase-conjugated secondary antibody (1 : 50, Servicebio, Wuhan, China).

### 2.14. Immunofluorescence (IF) Staining

The spleen tissues were fixed with acetone. IF staining was performed using the primary antibodies anti-CD4 (1 : 200, Servicebio, Wuhan, China) and anti-Cxcl7 (1 : 2000, Abcam, Cambridge, UK) and secondary antibody conjugated to FITC (1 : 500, Servicebio, Wuhan, China) in weak light conditions. Nuclei were stained with DAPI (Servicebio, Wuhan, China). With confocal microscopy, immunostained slides were imaged.

### 2.15. Statistical Analysis

DEGs were calculated and identified by the DESeq2 R package [26]. Significance was calculated by the hypothesis-testing probability (*p* value), as well as tested and corrected by the Benjamini-Hochburg method (also known as the FDR value). Significant DEGs were defined to meet the following two requirements: (i) more than twofold change and (ii) *p*adj < 0.05. The fold expression of each gene on CD4^+^T cells was calculated by comparing to the read counts against the standardized control. For the sake of convenience, log_2_ scale of fold change was adopted. Log_2_(fold change) > 1 means over twofold change. The experimental data of this study were obtained by more than three replicates. For other comparisons in this study, differences between groups were calculated by Student's *t*-test and ANOVA. *p* < 0.05 was considered significant. Differences between groups were analyzed by using the GraphPad Prism 7 software.

## 3. Result

### 3.1. Detect RNA Sequence Information of CD4^+^T Cells in Target Organs of HSCT Mice

**Figure 1:**
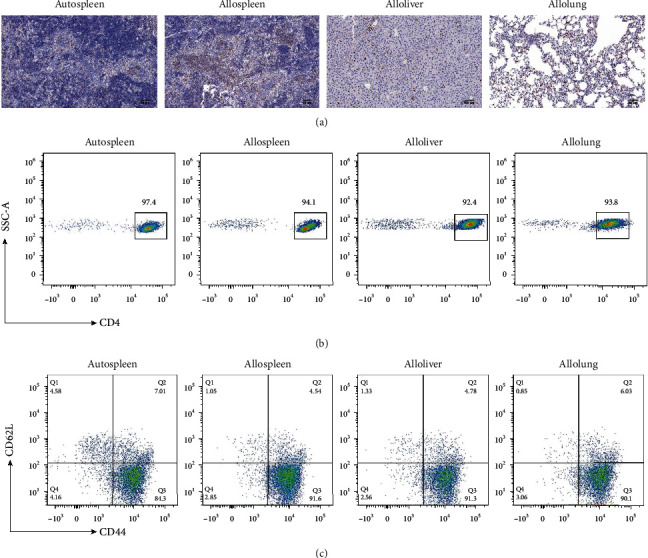
The ratio of the effector CD4^+^T cells. (a) The organs stained with antibodies of CD4. (b) Percent of CD4^+^T cells in autospleen, allospleen, alloliver, and allolung. (c) Percent of the effector CD4^+^T cells (CD4^+^CD44^+^CD62L^low^) in autospleen, allospleen, alloliver, and allolung. The effector CD4^+^T cells (CD4^+^CD44^+^CD62L^low^) were detected by flow cytometry.

The samples of target organs were excised on the 7th day after transplantation of and infiltration by CD4^+^T cells (Figure 1(a)). CD4^+^T in different organs of Balb/c recipient mice had >92% purity (Figure 1(b)). CD44^+^CD62L^low^ effector cells were about 90% in allo-HSCT mice (Figure 1(c)). Clean reads that met the quality control criteria of preprocessing constituted >96% of all reads in all samples (Figure S1A). The percentage of reads mapped in the exon region was above 94% (Figure S1B). The error rate of all samples was less than 0.1% (Figure S1C). For chain-specific libraries, the contents of A/T and G/C were almost the same (Figure S1D). Boxplots of the expression levels of all genes revealed no sample errors and outliers in Figure S2A. The correlation coefficient *R*^2^ and PCA analysis indicated the high comparability between groups (Figure S2B–S2C). These results indicated the high quality of our data with great replicability within groups and comparability between groups.

### 3.2. DEG Identification of CD4^+^T Cells from HSCT Mice

**Figure 2:**
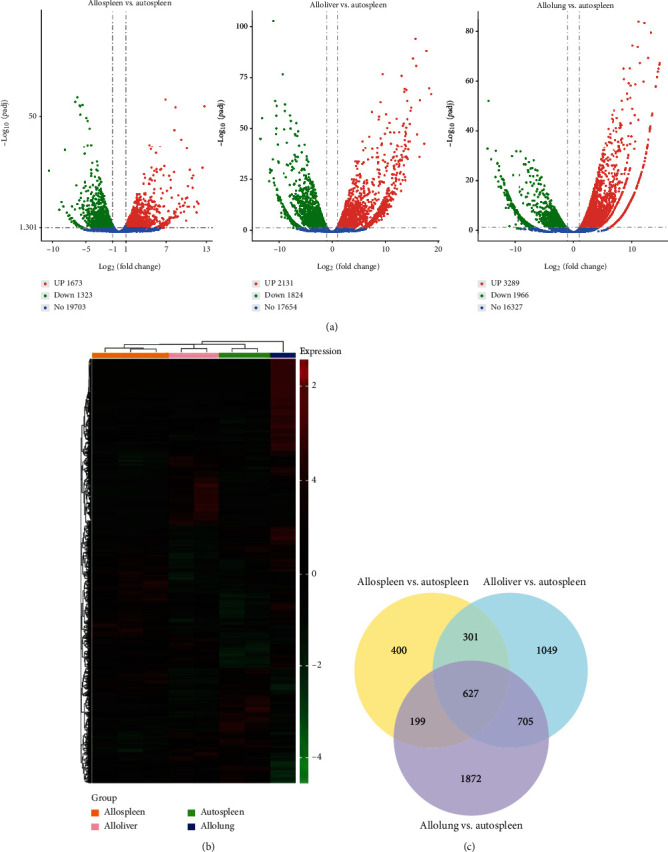
The DEGs among the spleen, liver, and lung in auto-HSCT and allo-HSCT aGVHD mouse models. (a) Volcano map of DEGs. Log_2_(fold change > 1) and *p*adj < 0.05 were considered significant. (b) The heat map of DEGs. Each column represents a sample, each row represents a gene, and different expression multiples are represented by different colors. The red blocks are for high expression genes, while the green blocks are for low expression genes. (c) Venn of DEGs. DEGs: differentially expressed genes.

In order to detect the impact of a xenoantigen and the intertissue microenvironment on donor CD4^+^T, the spleen CD4^+^T cells of allogeneic transplanted mice without the influence of the xenoantigen and microenvironment were regarded as the control group, and CD4^+^T cells from different organs of allogeneic transplanted mice affected by the xenoantigen and microenvironment were analyzed as an experimental group. The DEGs and samples were analyzed by clustering analysis (Figure 2(b)). The results indicated that genes with similar expression patterns were functionally correlated. Based on *p*adj < 0.05 and log_2_(fold change) > 1 shown in the volcano map (Figure 2(a)), a total of 2996 (1673 upregulated and 1323 downregulated), 3955 (2131 upregulated and 1824 downregulated), and 5255 (3289 upregulated and 1966 downregulated) DEGs were identified in allospleen vs. autospleen, alloliver vs. autospleen, and allolung vs. autospleen. We further explored the group-specific and overlapped DEGs in these groups. There were in total 627 overlapped DEGs in the autospleen, allospleen, allolung, and alloliver (Figure 2(c)).

### 3.3. Analysis of Immune-Related Pathways in HSCT Mice

**Figure 3:**
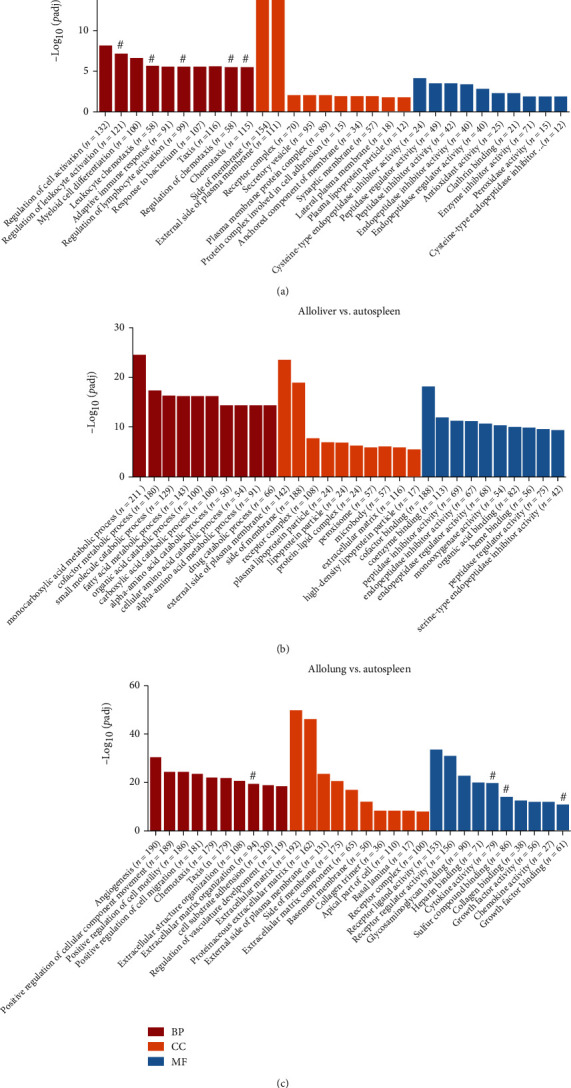
Enriched Gene Ontology (GO) terms. (a) The top 10 biological process (BP), cellular component (CC), and molecular function (MF) terms between allospleen and autospleen. (b) The top 10 BP, CC, and MF terms between alloliver and autospleen. (c) The top 10 BP, CC, and MF terms between allolung and autospleen. # for GO terms associated with immunoregulation.

Gene functions of DEGs were annotated and classified from three aspects: biological process (BP), cellular component (CC), and molecular function (MF). After a comparative study, data discrepancies enriched in DEGs are shown, respectively: 380 BP terms, 14 CC terms, and 17 MF terms in allospleen vs. autospleen; 851 BP terms, 48 CC terms, and 141 MF terms in alloliver vs. autospleen; and 1339 terms, 47 CC terms, and 93 MF terms in allolung vs. autospleen. The top 10 GO terms are presented in Figure 3. It should be noted that 6 BP terms (regulation of leukocyte activation, leukocyte chemotaxis, regulation of lymphocyte activation, regulation of chemotaxis, chemotaxis, and leukocyte migration) and 3 MF terms (cytokine activity, chemokine activity, and cytokine receptor binding) were distinguished to be associated with immunoregulation (Figure 3).

**Figure 4:**
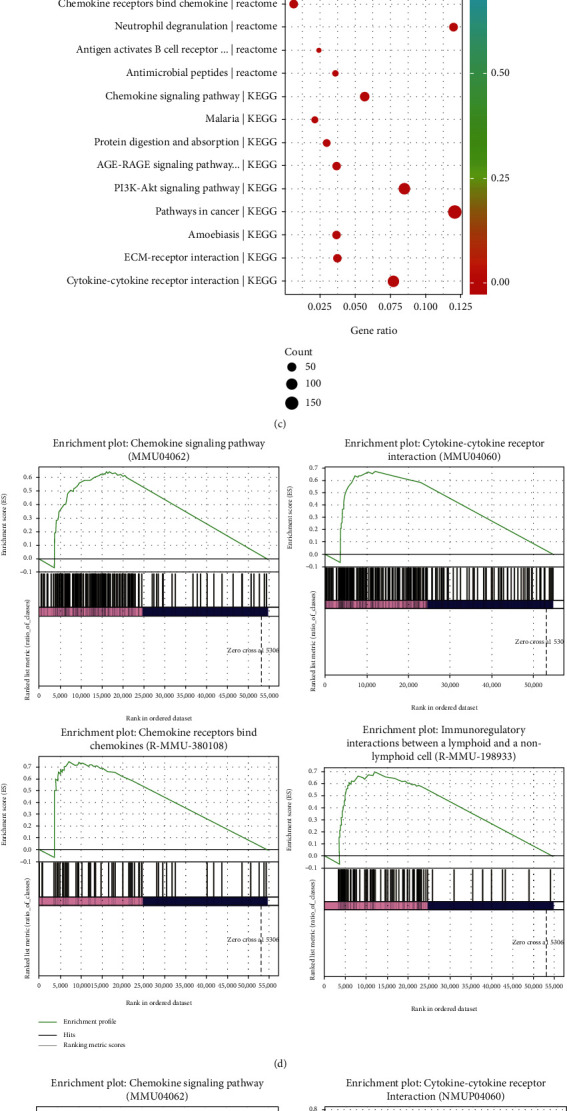
Enriched Kyoto Encyclopedia of Genes and Genomes (KEGG) and Reactome pathway terms. The top 10 KEGG and Reactome terms between allospleen and autospleen (a), alloliver and autospleen (b), and allolung and autospleen (c). The immune-related KEGG and Reactome pathways were analyzed using GSEA assays between allospleen and autospleen (d), alloliver and autospleen (e), and allolung and autospleen (f). The size of the dots represents the number of enrichment genes in the pathway. The different colors of the dots indicate the different *p* values.

The top 10 most significantly enriched pathways are shown using KEGG and Reactome (Figures 4(a)–4(c)). Gene set enrichment analysis (GSEA) revealed 2 KEGG pathways (chemokine signaling pathway, cytokine and cytokine receptor interaction) and 2 Reactome pathways (chemokine receptors binding chemokines, immunoregulatory interactions between a lymphoid and a nonlymphoid cell). The four pathways mentioned above belonged to immunoregulation in GVHD (Figures 4(d)–4(f)).

### 3.4. Potential Immunoregulatory Genes in DEGs

To gain insight into the relationships between DEGs, we constructed a PPI network with high confidence interactions using STRING and Cytoscape. The top 10 hub genes from a total of 60 hub genes from each group identified by the CytoHubba plugin are listed in Table 2. 11 out of the 60 hub genes associated with immunoregulation have not been studied in GVHD, including *Rgs1*, *Fpr2*, *Ppbp* (*Cxcl7*), *S1pr3*, *Npy*, *Hebp1*, *Kng1*, *Plg*, *Cxcl16*, *Ccl9*, and *Cxcl3*. The PPI network among the 11 genes was constructed (Figure S3A). According to MCC, *Cxcl16*, *Kng1*, and *Cxcl7* were considered as more critical hub genes (Figure S3B). Moreover, we applied the MCODE plugin to analyze PPIs to predict significant protein complexes. One significant module was identified in the DEG regulatory network, which contained 8 genes, including *Cxcl3*, *Npy*, *Cxcl16*, *Kng1*, *Ccl9*, *S1pr3*, *Fpr2*, and *Cxcl7* (Figure S3C).

### 3.5. Validation of the Sequence Results *In Vitro*

**Figure 5:**
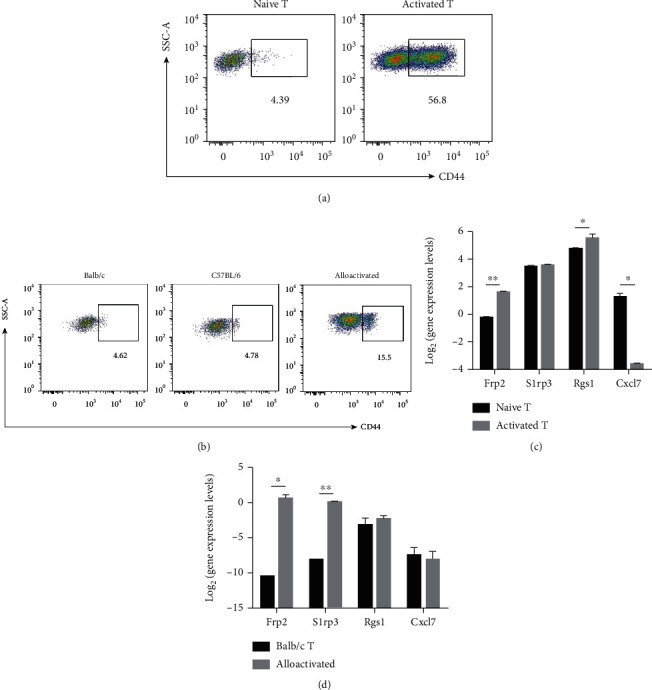
The verification of the expression of hub genes in vitro. (a) The percent of activated CD4^+^T cells in naive and activated T cells by induction of anti-CD3/CD28 antibodies. (b) The percent of the activated CD4^+^T cells in Balb/c, C57BL/6, and alloactivated T cells after MLR. The activated CD4^+^T cells (CD4^+^CD44^+^) were detected by flow cytometry analysis. (c) The expression levels of genes from activated T cells were detected. (d) The expression levels of genes from FACS-sorted activated CD4^+^T cells were detected. The *y*-axis is the log2 scale of the expression level of hub genes. Each experiment was performed at least three times, and the results were presented as mean ± SD.

To confirm the credibility of the DEGs identified from RNA-Seq, we performed qRT-PCR to verify the expression level of the DEGs. The CD4^+^T cells activated by MLR and cytokines (Figures 5(a) and 5(b)) were obtained by FACS sorting. Four related hub genes (*Rgs1*, *Fpr2*, *Cxcl7*, and *S1pr3*) were verified by activated CD4^+^T cells through MLR and cytokine stimulation (Figures 5(c) and 5(d)). These genes showed consistent results as those found in RNA-Seq.

### 3.6. Validation of the Sequence Results *In Vivo*

**Figure 6:**
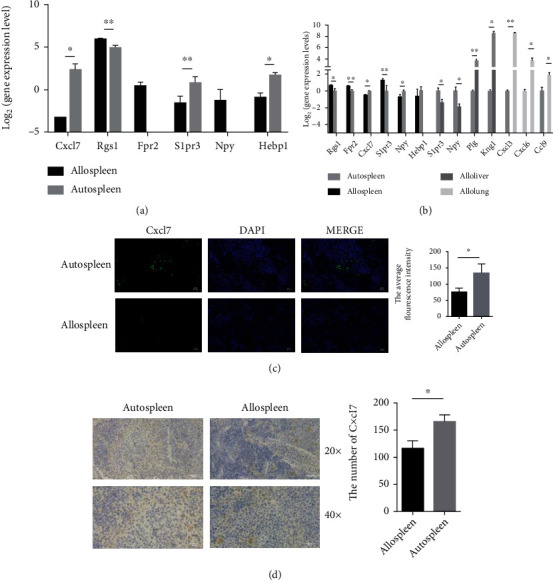
The verification of the expression of hub genes in vivo. (a) The hub genes from allospleen and autospleen were verified in GSE126518. (b) The hub genes were verified from different tissues by qRT-PCR. CXCL7 deposition was detected in target areas of the spleen in aGVHD mouse animals. (c) Quantification of the histology staining shown. At the left is one representative section per group. At the right is the number of CXCL7 per scale in the spleen. CXCL7 were detected by colocalization of CXCL7 (green) and DAPI (blue). CXCL7 deposition was quantified on a 0-3 scale to determine the amount of antibodies in the tissues. (d) Representative spleen histology staining revealing the expression of CXCL7 (brown) 7 d after HSCT in auto-HSCT and allo-HSCT mice. Cells were stained with CXCL7 (brown). Bars: 50 or 200 *μ*m. ^∗^*p* < 0.05 and ^∗∗^*p* < 0.01. The *y*-axis is the log2 scale of the expression level of hub genes. Each experiment was performed at least three times, and the results were presented as mean ± SD.

The validation data GSE126518 obtained from the GEO database was further used to verify the results (Figure 6(a)). We also selected the 11 novel hub genes that were significantly differentially expressed among the livers, lungs and spleens. As shown in Figure 6(b), most of the genes showed consistent results as those found in RNA-Seq. The protein levels of CXCL16, KNG1, and CXCL7 were verified by histological research and immunofluorescence staining. The results showed that the expression level of CXCL7 in the autospleen was higher than that in the allospleen (Figures 6(c) and 6(d)). However, the KNG1 protein and the CXCL16 protein were almost undetectable in tissues between the autospleen and allospleen, and there was no statistical difference about the expression level of KNG1 and CXCL16 between two groups (the data was not shown). Moreover, it was found that the CXCL7 expression level was also elevated in the autoliver and autolung (Figure S4). In conclusion, the results basically verified the reliability of the result.

## 4. Discussion

Acute GVHD is induced by numerous graft-derived immunocytes, including the naive CD4^+^T cells from donors, which can differentiate and exert cytotoxicity on multiple organ systems of the allo-HSCT recipients [4]. However, it was uncertain whether effector CD4^+^T expressed differently among different organs. RNA-Seq, a method of comprehensive transcriptome profiling, generates a deep sequencing data for the direct quantification of the transcripts by next-generation sequencing (NGS) technologies [27]. With integrative transcriptome analysis, the study is aimed at exploring potential molecular mechanisms involved in CD4^+^T cells in different target organs sampled from auto-HSCT and allo-HSCT aGVHD mouse models. We identified significant DEGs and critical enriched pathways, including the chemokine receptor binding chemokine pathway, immunoregulatory interactions between a lymphoid and a nonlymphoid cell, the cytokine and cytokine receptor interaction pathway, and the chemokine signaling pathway. 11 novel genes that likely participated in the immunoregulation of the pathogenesis of aGVHD were also identified. In the PPI network, significant protein complexes containing DEG regulators were detected.

RNA-Seq was not free of biases. Results could be affected by differences in fragment size, transcript length, and differences in GC content [28]. In this study, to ensure a high quality and creditability of the data and results, we collected CD4^+^T cells (>92%) and CD44^+^CD62L^low^ effector cells (about 90%) with high purity in allo-HSCT mice, and applied quality control filters in the preprocessing of the raw read before DEG identification. Boxplots, correlation heat maps, and PCA plots of the gene expression FPKM values provided further evidence for the high quality and creditability of our data.

Organs have unique structures, physiological functions, and tissue microenvironments, thereby forming local immune environments that may be closely related to the occurrence and development of aGVHD associated with the organs. The spleen is the largest peripheral lymphatic organ in the human body, harboring various types of lymphocytes, mainly B and T cells. These cells in the spleen exert immune responses when the body is invaded by pathogens. However, there are few studies on the local immune environments of target organs in aGVHD. To further explore the immunopathological mechanism of GVHD, we focused on DEGs of the spleen, liver, and lung hub genes with immunoregulatory functions in a murine model of allogeneic HSCT compared with splenic CD4^+^T cells from mice with autologous transplantation.

We identified 627 overlapped DEGs and 400, 1872, and 1049 organ-specific DEGs by CD4^+^T in the allospleen, allolung, and alloliver compared to the autospleen. Many DEGs were found associated with GVHD in previous studies, such as CD34 and CD33 [29, 30]. We also identified many novel DEGs that were firstly reported to be associated with aGVHD in our study. Pathway enrichment analysis and PPI networks are helpful ways to extract the main information from a large list of genes, often used in transcriptome analysis. Due to the effect on the relevant pathway and the regulation of other genes, hub genes in PPI networks provide promising targets and research hotspots in a biophysiological and pathological developmental process study. We identified 60 hub genes in PPI networks of DEGs, including 11 novel genes that had not been reported in previous GVHD studies. 11 novel genes were later confirmed with qRT-PCR.

We observed that a significant portion of the hub genes were chemokine genes, especially the upregulated DEGs in the spleens and lungs from allogenic recipient mice. Reactome pathway analysis of the DEGs also highlighted the importance of the chemokine receptor binding chemokine pathway. One main function of chemokines is to induce immune cells to enter the site of infection during the immune response [31]. In the aGVHD model, the activated effector T cells migrate to target tissues and organs to recruit more effector cells, resulting in the damage of tissues and organs. The role of chemokines in GVHD has been studied by our team and many other researches [13, 14]. We found that the expression of *Cxcl3* was consistent with the enrichment of the pathway. *Cxcl3* increases the activity toward human granulocytes [32]. Other nonchemokine genes, such as *Rgs1* and *Fpr2*, can increase the response of chemokines, regulate inflammatory mediators, and enhance immune regulation properties [33, 34]. In this study, we identified more chemokine genes that had not been previously reported in GVHD, especially *Cxcl7*, *Kng1*, and *Cxcl16* which may be the study focus in future studies.

Our study found that cytokine and cytokine receptor interaction pathways were significantly enriched in DEGs by KEGG pathway analysis. Cytokines had important immunoregulatory functions during immune responses. At the early stage of GVHD, cytokines recruited various subsets of immune cells to an immune context. Our study found increased expression of *Npy* in the spleen of auto-HSCT. *Npy* can regulate the expression of Interleukin-4 (IL-4) [35], a leukocyte chemotactic-activating cytokine that guided the differentiation of naive CD4^+^T cells into Th2 cells [36]. The high expression of *Npy* from auto-HSCT promoted DCs to polarize Th2 differentiation by upregulating IL-10 [37]. Moreover, *Npy* could decrease the production and release of IL-6 from splenic macrophages [38], which may be related to the reduction of the severity of the disease caused by the inhibition of cytokine release.

Reactome enriched pathway analysis also discovered immunoregulatory interactions between a lymphoid and nonlymphoid cell pathway from the DEGs. Among the hub genes, the *Plg* gene, a liver-specific gene [39], was found highly expressed in CD4^+^T cells from the liver of aGVHD model. Mitchell et al. [40] revealed that the *Plg* promotes monocyte proliferation in inflammation response. Its function in aGVHD, however, is unknown. Epithelial cells can collaborate with immune cells to regulate innate and adaptive responses in several tissue types [41]. It is possible that genes like *Plg* might be involved in the immunoregulatory interaction of the lymphoid and nonlymphoid cells. Regulation of these genes with the characteristics of immune regulation and tissue-specific expression could be used to decrease the influence of activated CD4^+^T cells to infiltrate and damage organs in the future.

## 5. Conclusion

In conclusion, we identified 11 novel hub genes related to aGVHD. Our findings enhanced the understanding of the mechanism of aGVHD target organ damage and provided a prioritized list of genes for future studies. More such studies will eventually provide evidences for preclinical trials using cellular and animal models and accelerate the development of new methods for clinical therapies.

## Figures and Tables

**Table 1:** Primers for 11 candidate immunoregulatory genes and *Gapdh.*

Gene	Forward primer (5′ → 3′)	Reverse primer (5′ → 3′)	Product length
*Cxcl7*	CTCACGTTGTTCCCTCCTGG	TGGGTCCATGCCATCAGATT	192
*S1pr3*	TCAACACTCTTCCCGCAGTC	CCCGGAGAGTGTCATTTCCC	192
*Npy*	TAACAAGCGAATGGGGCTGT	TGATGTAGTGTCGCAGAGCG	61
*Hebp1*	CCTGTCTACTCACGCGCTTA	GGAGACATCTTCCTTGCCCC	180
*Rgs1*	TGCCAACCAGACAGGTCAAA	TTCACAGCATCTGAATGCACAA	171
*Fpr2*	TTCATGGGCCAGGACTTTCG	CACAGACTTCATGGGGCCTT	155
*Cxcl16*	GGGCTTTGGACCCTTGTCTC	GATCCAAAGTACCCTGCGGT	152
*Ccl9*	CAGGCCGGGCATCATCTTTA	AGTAGCTGGCAGTTCACACC	165
*Cxcl3*	CTGCACCCAGACAGAAGTCAT	CCGTTGGGATGGATCGCTTT	198
*Plg*	TCCGTGGGTTGGATGTTCAG	ATTGTCCGGTCAGCAACCAT	163
*Kng1*	TCCCGACTGTGAAATGCCAA	CCCAGTGTCATATGGTGGGG	164
*Gapdh*	CTCATGACCACAGTCCATGC	CACATTGGGGGTAGGAACAC	201

**Table 2:** The top 10 up- and downregulated hub genes identified in CD4^+^T cells from each organ.

Group		Hub gene
Allospleen vs. autospleen	Upregulated	*Cxcr2*, *Ccr1*, *Ccl5*, *Rgs1*, *Cxcl10*, *Cxcr5*, *Cxcr6*, *Cxcl2*, *Cxcl9*, and *Fpr2*
	Downregulated	*Ccr7*, *Cxcl7*, *Ccr6*, *Ccr4*, *Sstr2*, *Penk*, *S1pr3*, *Npy*, *Hebp1*, and *Flt3*
Alloliver vs. autospleen	Upregulated	*Kng1*, *Plg*, *Rdh7*, *Cps1*, *Serpinc1*, *Apof*, *Uox*, *Fga*, *Apoc3*, and *Hpx*
	Downregulated	*Ccr7*, *Cxcr4*, *Ccr3*, *Ccr4*, *Htr1b*, *Ccr6*, *Cxcl7*, *S1pr3*, *Npy*, and *Ptger3*
Allolung vs. autospleen	Upregulated	*C3*, *Cxcl16*, *Ccl9*, *Ccr1*, *Cxcr2*, *Cxcl5*, *Cxcl3*, *Cxcl1*, *Ccr8*, and *Cxcl10*
	Downregulated	*Ccnb1*, *Cdc20*, *Aurkb*, *Aurka*, *Plk1*, *Bub1b*, *Mcm2*, *Cdc6*, *Mcm5*, and *Cdk1*

## Data Availability

All the data of this study are openly available upon request.

## Additional Points

*Highlights.* (1) Identify differentially expressed genes of CD4+lymphocyte in comparison to tissues by major histocompatibility complex- (MHC-) mismatched and MHC-matched murine BMT models. (2) Identify 11 novel immunoregulatory genes that were not reported to be associated with aGVHD. (3) Identify 4 immunoregulatory pathways (cytokine and cytokine receptor interaction, chemokine receptors binding chemokines, and lymphoid and a nonlymphoid cells) in aGVHD. (4) CXCL7 was highly expressed in allo-HSCT

## Supplementary Materials

Supplementary Materials Figure S1: the quality of sequencing data. (A) Classification of raw reads: “Clean Reads” for the percent of clean reads; “Adapter Related” for the percent of reads with an adapter; “Containing*N*” for the percent of reads with *N* bases; and “Low Quality” for percent of reads with low sequencing quality. (B) Percent of genome regions: “exon” for the number of exon reads and the percent to the clean reads; “intron” for the number of intron reads and the percent to the clean reads; “intergenic” for the number of intergenic regions and the percent to the clean reads. (C) Error rate distribution along reads. (D) Base content along reads. The base distribution of two reads of double-terminal sequencing sequence was presented on the left and right sides. Figure S2: The quantitative analysis of sequencing data. (A) The gene expression distribution. FPKM corrected for sequencing depth and gene length. The maximum, upper quartile median, lower quartile, and minimum (top to down) values of gene expression in each sample are presented. (B) The Pearson correlation between samples. The *R*^2^ value and their expression pattern had a positive correlation. (C) Principal component analysis (PCA) used the calculation method of linear algebra to reduce the dimension and extract the principal components of tens of thousands of gene variables. Figure S3: bioinformatics analysis of protein quantitative identification in allospleen, autospleen, allolung, and alloliver. (A) PPI network of genes related to immunoregulation. *Ppbp* was the same as *Cxcl7*. (B) The hub genes were highlighted in red. (C) The module comprises 8 nodes and 28 edges. Figure S4: the expression of Cxcl7 in target organs. (A–B) Quantification of the histology staining shown in the lung and liver. At the left is one representative section per group. At the right is the average fluorescence intensity of Cxcl7 per scale in the liver and lung, respectively. Cxcl7 were detected by colocalization of Cxcl7 (green) and DAPI (blue). Cxcl7 deposition was quantified on a 0-3 scale to determine the amount of antibody in the tissues. (C–D) Representative lung and liver histology staining revealing the expression of Cxcl7 (brown) 7 d after HSCT in auto-HSCT and allo-HSCT mice. Cells were stained with Cxcl7 (brown). At the left is one representative section per group. At the right is the number of Cxcl7 per scale in the liver and lung, respectively. Bars: 50, 100, and 200 *μ*m.
