# Supplementary material for: The Potential Genes Mediate the Pathogenicity of Allogeneic CD4+T Cell in aGVHD Mouse Model
Source: Biomed Res Int. 2021 May 7;2021:9958745. doi: 10.1155/2021/9958745 (PMC8121574; doi:10.1155/2021/9958745)

Fig. S1 The quality of sequencing data. (A) Classification of raw reads. Clean reads for the percent of clean reads; Adapter related for the percent of reads with adapter; Containing N for the percent of reads with N bases; Low quality for percent of reads with low sequencing quality. (B) Percent of genome regions. Exon for the number of exons reads and the percent to the clean reads; intron for the number of intron reads and the percent to the clean reads; intergenic for the number of intergenic regions and the percent to the clean reads. (C) Error rate distribution along reads. (D) Bases content along reads. The base distribution of two reads of double-terminal sequencing sequence was presented on the left and right sides.


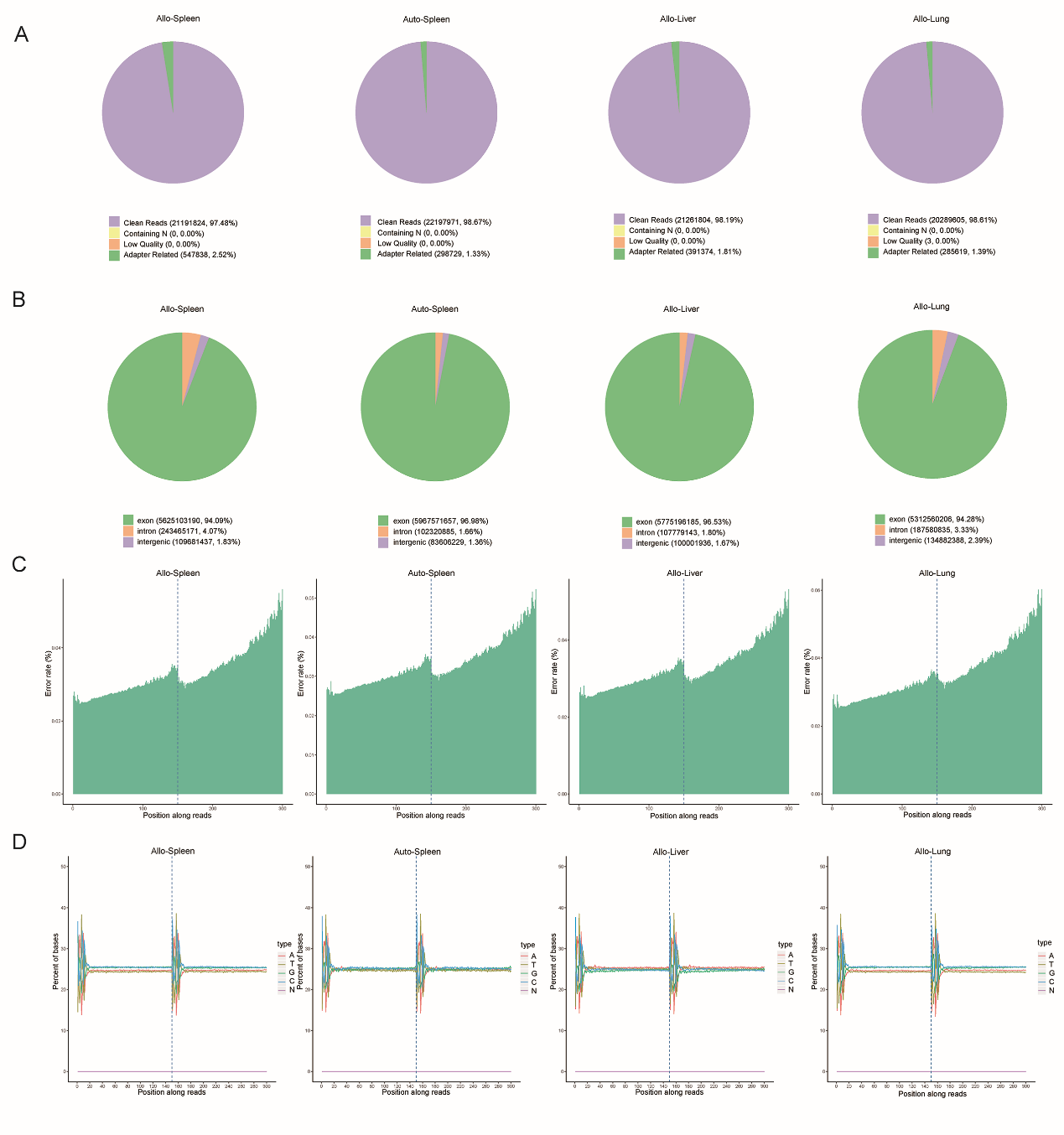


Fig. S2 The quantitative analysis of sequencing data. (A) The gene expression distribution. FPKM corrected for sequencing depth and gene length. The maximum, upper quartile median, lower quartile, and minimum (top to down) values of gene expression in each sample were presented. (B) The Pearson correlation between samples. The R^2^ value and their expression pattern were positive correlation. (C) Principal component analysis. PCA used the calculation method of linear algebra to reduce the dimension and extract the principal components of tens of thousands of gene variables.


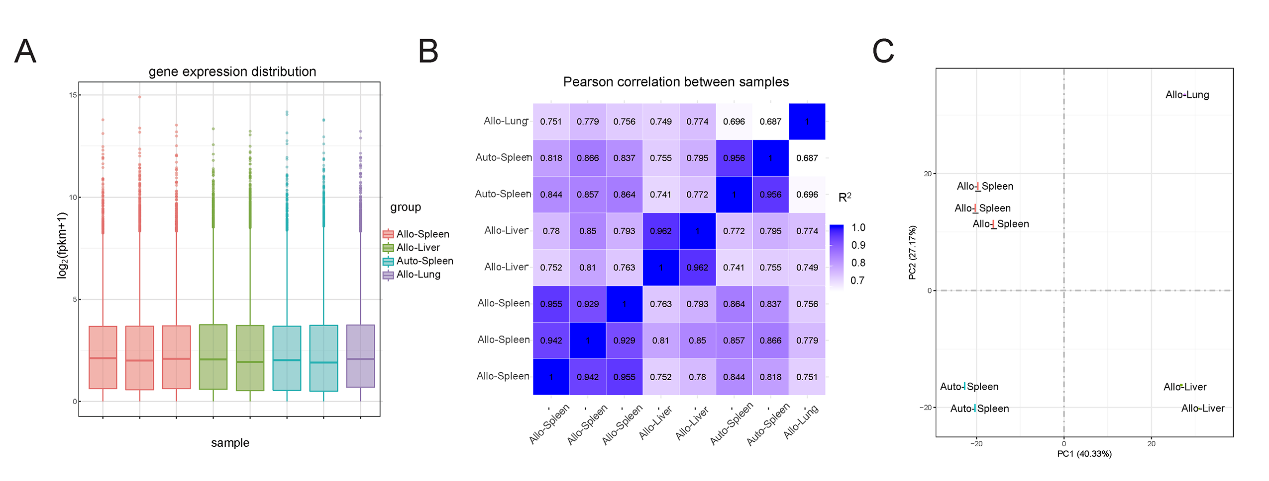


Fig. S3 Bioinformatics analysis of protein quantitative identification in Allo-Spleen, Auto-Spleen, Allo-Lung and Allo-Liver. (A) PPI network of genes related to immunoregulation. *Ppbp* was the same as *Cxcl7*. (B) The hub genes were highlighted in red. (C) The module comprises 8 nodes and 28 edges.


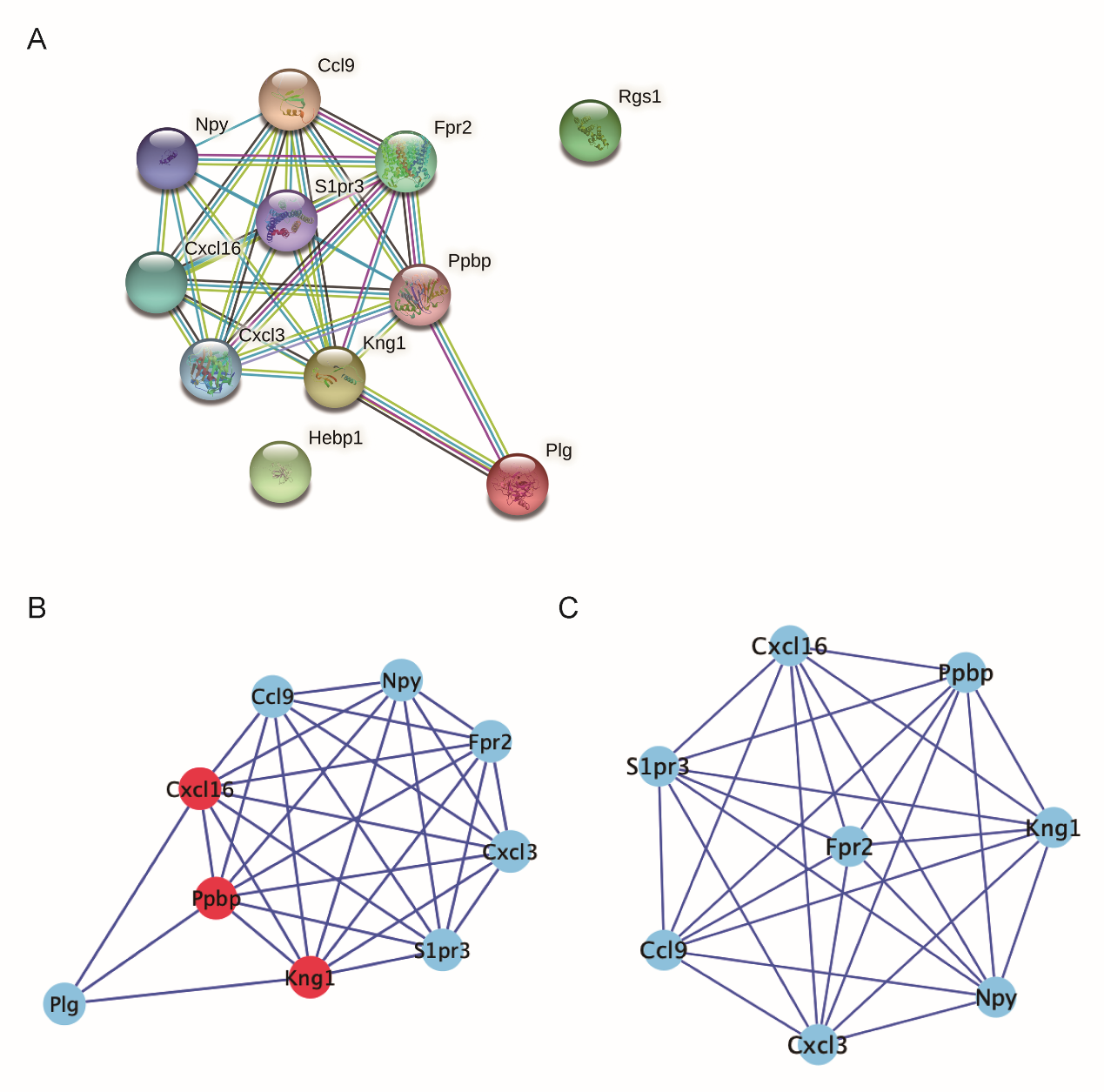


Fig. S4 The expression of Cxcl7 in target organs. (A-B) Quantification of the histology staining shown in lung and liver. The left was one representative section per group. The right was the average fluorescence intensity of Cxcl7 per scale in the liver and lung respectively. Cxcl7 were detected by colocalization of Cxcl7 (green) and dapi(blue). Cxcl7 deposition was quantified on a 0-3 scale to determine the amount of antibody in the tissues. (C-D) Representative lung and liver histology staining revealing the expression of Cxcl7 (brown) 7 d after HSCT in Auto-HSCT and Allo-HSCT mice. Cells were stained with Cxcl7 (brown). The left was one representative section per group. The right was the number of Cxcl7 per scale in the liver and lung respectively. Bars, 50, and 100, 200 μm.


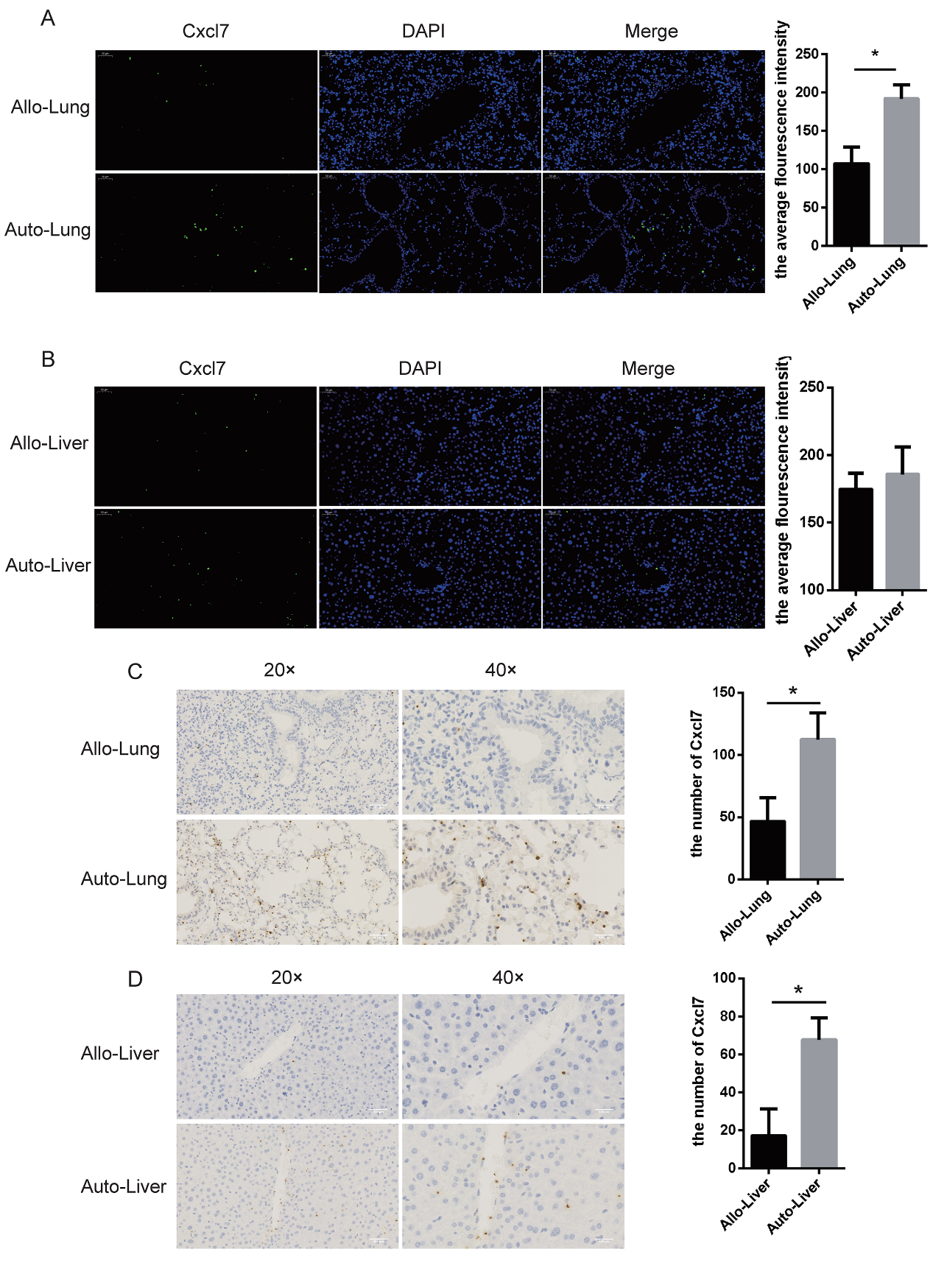

Supplement: Supplementary Materials — Figure S1: the quality of sequencing data. (A) Classification of raw reads: “Clean Reads” for the percent of clean reads; “Adapter Related” for the percent of reads with an adapter; “ContainingN” for the percent of reads with N bases; and “Low Quality” for percent of reads with low sequencing quality. (B) Percent of genome regions: “exon” for the number of exon reads and the percent to the clean reads; “intron” for the number of intron reads and the percent to the clean reads; “intergenic” for the number of intergenic regions and the percent to the clean reads. (C) Error rate distribution along reads. (D) Base content along reads. The base distribution of two reads of double-terminal sequencing sequence was presented on the left and right sides. Figure S2: The quantitative analysis of sequencing data. (A) The gene expression distribution. FPKM corrected for sequencing depth and gene length. The maximum, upper quartile median, lower quartile, and minimum (top to down) values of gene expression in each sample are presented. (B) The Pearson correlation between samples. The R2 value and their expression pattern had a positive correlation. (C) Principal component analysis (PCA) used the calculation method of linear algebra to reduce the dimension and extract the principal components of tens of thousands of gene variables. Figure S3: bioinformatics analysis of protein quantitative identification in allospleen, autospleen, allolung, and alloliver. (A) PPI network of genes related to immunoregulation. Ppbp was the same as Cxcl7. (B) The hub genes were highlighted in red. (C) The module comprises 8 nodes and 28 edges. Figure S4: the expression of Cxcl7 in target organs. (A–B) Quantification of the histology staining shown in the lung and liver. At the left is one representative section per group. At the right is the average fluorescence intensity of Cxcl7 per scale in the liver and lung, respectively. Cxcl7 were detected by colocalization of Cxcl7 (green) and DAPI (blue). Cx [file 9958745.f1.docx]
